# Quantitative Evaluation of Proliferative Potential Using Flow Cytometry Reveals Intratumoral Heterogeneity and Its Relevance to Tumor Characteristics in Vestibular Schwannomas

**DOI:** 10.3390/curroncol29030134

**Published:** 2022-03-03

**Authors:** Soichi Oya, Shinsuke Yoshida, Shunya Hanakita, Mizuho Inoue

**Affiliations:** Department of Neurosurgery, Saitama Medical Center, Saitama Medical University, Saitama 350-8550, Japan; sinyosida@msn.com (S.Y.); hanakita-s@umin.ac.jp (S.H.); zumi.eunoi@gmail.com (M.I.)

**Keywords:** flow cytometry, heterogeneity, MIB-1, proliferative potential, vestibular schwannoma

## Abstract

This study sought to explore the existence and clinical significance of intratumoral heterogeneity of proliferative potential in vestibular schwannoma (VS). Rapid intraoperative flow cytometry was utilized with raw samples to measure the proliferative ability of VS. The proliferation index (PI) was defined as the ratio of the number of cells with greater than normal DNA content to the total number of cells. A total of 66 specimens (26 from the intrameatal portion and 40 from the cisternal portion) were obtained from 34 patients with VS. There was a moderate correlation between the PI and MIB-1 labelling index values (R = 0.57, *p* < 0.0001). In contrast, the patterns of heterogeneity, represented by the proportion of intrameatal PI to cisternal PI, were associated with tumor size (*p* = 0.03). In addition, preoperative hearing tended to be poor in cases where the intrameatal PI was higher than the cisternal PI (*p* = 0.06). Our data demonstrated the presence of intratumoral heterogeneity of proliferative potential in VS and its relationship with tumor characteristics. The results of this study may advocate the resection of the intrameatal portion of large VSs treated with planned subtotal resection, especially in cases of poor preoperative hearing function.

## 1. Introduction

In the treatment of brain tumors, it is vital to achieve the best balance between long-term tumor control and functional preservation. Specifically, for histologically benign tumors such as meningiomas and schwannomas, surgical risks should be carefully weighed against the benefits of excellent long-term tumor control and positive functional outcomes. Owing to the high proliferation rate and resistance to radiation therapy of a subset of meningiomas, radical resection is sometimes required. On the other hand, vestibular schwannomas (VSs) are histologically more benign and respond better to gamma knife radiosurgery (GKRS) than meningiomas, with an extremely low rate of malignant transformation after radiation [1]. For small- to medium-sized previously untreated VS, GKRS provided a tumor control rate of 92–98% during a follow-up of 10 years with a facial nerve complication rate of 1% or lower [2,3,4]. Therefore, treatment decisions for VS have been trending toward functional preservation rather than resection [5,6]. Given this shift in treatment strategy, redefinition of the role of surgical resection has been a target of debate in VS treatment. Currently, small- to medium-sized VSs are often treated conservatively or with radiosurgery, whereas large VSs are frequently treated with subtotal resection (STR) followed by adjunctive radiosurgery for the best functional preservation of the facial nerve [7]. However, a subset of patients treated with STR eventually experience tumor recurrence that requires additional treatment [8,9]. Whether surgeons can lower the risk of recurrence after STR by further understanding the biological characteristics of VS remains to be elucidated. Interestingly, Breshears, J.D. et al. recently reported that residual tumors in the internal auditory meatus increased the risk of shorter progression-free survival [10]. Their data raise the question of whether regional differences of proliferative potential exist in VS. Intraoperative flow cytometry (iFC) has been utilized to quantitatively assess the proliferative ability of brain tumor cells [11,12,13,14,15]. In the present study, we aimed to explore whether intratumoral heterogeneity of proliferative potential exists in VS and, if so, whether it is correlated with the risk of recurrence and clinical features.

## 2. Materials and Methods

### 2.1. Patient Population and Tumor Characteristics

This study was approved by the institutional review board (No. 2542). Patients with VS who underwent surgical resection between August 2015 and October 2021 were enrolled in this study. Written informed consent was obtained from all patients. Patients were excluded if they had recurrent or previously irradiated VS. In all, surgery was performed for 34 patients who met the above criteria. According to magnetic resonance (MR) imaging findings, tumors were classified as solid or cystic. When the cystic portion occupied >50% of the tumor on MR images, the tumor was characterized as cystic. Tumor size was determined by measuring the maximum size of the cisternal portion on MR axial images. Hearing function for all patients was assessed preoperatively by an otologist using the Gardner–Robertson (GR) hearing classification scale [16]. In this study, GR classes I and II were considered to indicate good hearing function, whereas classes III–V were considered to indicate poor hearing function. Pre- and postoperative facial nerve function was evaluated using the House–Brackmann (HB) grading scale, and patients were regarded as having facial paresis when the HB grade was III or higher. Postoperative facial nerve function was assessed at the 6-month follow-up for patients with facial nerve paresis. The same surgeon (S.O.) performed all tumor resections using the lateral suboccipital approach with facial nerve monitoring. Moreover, hearing preservation was attempted for selected patients with good hearing function who wished to preserve it. Our surgical strategy was to prioritize facial nerve function over gross total resection by intraoperative facial nerve monitoring. During surgery, a 5-mm tumor specimen was obtained from the cisternal portions of the tumor. No tumor was located solely in the meatus in this study. For tumors larger than 2 cm, a same-sized specimen was also obtained from the intrameatal portion. For tumors larger than 3 cm, 3 specimens were obtained from each portion of the intrameatal portion and the center and peripheral areas of the cisternal portion. Each specimen was equally dichotomized for MIB-1 staining and iFC. The specimens obtained from each region were separately sent to the laboratory for iFC within an hour. Other radiological characteristics, such as the maximum size of the cisternal portion, cystic or solid classification, and the diameter of the meatus, were evaluated from the preoperative MR images. The size of the internal auditory meatus was measured using axial computed tomography (CT) images of the porus acusticus internus. Immediate postoperative MR images were used to classify the extent of resection as gross total resection, near-total resection, or subtotal resection in accordance with resection rates of 100%, 95–99%, and 95% or lower, respectively. All patients underwent follow-up MR imaging 3–6 months after surgery. Unless recurrence or regrowth was observed on serial MR imaging, follow-up imaging was performed annually thereafter.

### 2.2. iFC and Histological Analysis

**Figure 1:**
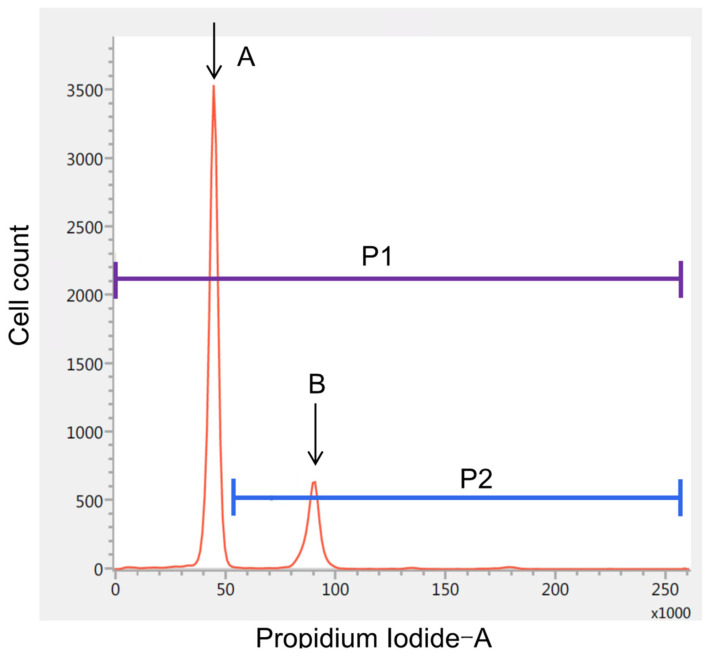
DNA ploidy analysis using intraoperative flow cytometry. The horizontal axis represents the intensity of propidium iodide fluorescence. Peak A indicates the cluster of G_0_G_1_-phase (diploid) cells, whereas Peak B represents that of tetraploid G_2_/M-phase cells. The proliferation index was defined as the ratio of the number of cells with greater-than-normal DNA content (P2) to the total number of cells (P1).

In addition to standard histological diagnosis, we used iFC to assess the proliferative potential of tumor cells. We followed the previously reported detailed procedure for iFC [11]. Briefly, an approximately 2 mm specimen obtained during surgery was immediately (within an hour) sent to the laboratory, where it was immersed in a microtube with a kit solution (DNA Peak; Nihon Koden Corporation, Tokyo, Japan). The specimen was then disrupted by repetitive pipetting for 200 s. Next, the homogenized sample was mixed with a surface-acting agent to stain the cell nuclei at room temperature. The suspension was filtered through a 50 µm nylon mesh, and its DNA content was measured using a BD FACSverse flow cytometer (Becton Dickinson Biosciences, Franklin Lakes, NJ, USA) to obtain the DNA histogram (Figure 1). Peak A indicated G_0_G_1_ phase (euploid) cells, and cells in the area to the left of peak A were the sum of sub-G_0_G_1_-phase cells, apoptotic cells, and debris. Peak B represented both aneuploid cells with an abnormally high number of chromosomes and G_2_/M-phase cells. Cells falling in the interval between peaks A and B were in the S phase. To investigate the proliferative potential of each tumor, the proliferation index (PI) was calculated as the ratio of the number of cells with greater-than-normal DNA content to the total number of cells. The actual time required for flow cytometry was approximately 10 min.

Before starting this study, we conducted a preliminary experiment to validate the quantitative capability of iFC. We divided the sample into 10 tubes after sufficient agitation and subsequently measured the PI. The mean and standard deviation were 9.11 and 0.76, respectively; thus, the 95% confidence interval (95% CI) was 8.57–9.65.

Pathological diagnosis was conducted at the Department of Pathology at Saitama Medical Center. The MIB-1 labelling index (LI) was calculated in a blind fashion using the most accurate LI method, focusing on areas of maximum density identified through visual analysis.

### 2.3. Statistical Analysis

Pearson’s product-moment correlation coefficient was used to evaluate the statistical significance of the correlation between the MIB-1 LI and PI. Fisher’s exact and Wilcoxon rank-sum tests were used to evaluate differences in demographic and clinical factors between groups. All analyses were performed using JMP 16.0.0 (SAS Institute, Cary, NC, USA).

## 3. Results

### 3.1. Patient Characteristics

A summary of patient demographics and tumor characteristics is presented in Table 1. After the inclusion and exclusion criteria were applied, 34 patients with VS who underwent surgery were enrolled in this study. From these patients, 66 samples were obtained from the intrameatal and cisternal portions. The mean age was 57.8 years (range, 16–78 years). A total of 21 patients were female (61.8%). The average size of the cisternal portion of the tumor was 32.3 mm (range, 14–55 mm). On MRI, 21 tumors (61.8%) were observed to be solid, while the rest were classified as cystic. Additionally, the mean size of the internal auditory meatus was 9.3 mm. Preoperative hearing function was reported as grade I in 6 patients, grade II in 3 patients, grade III in 4 patients, and grade V in 20 patients. One patient was born bilaterally deaf. The mean MIB-1 LI and PI were 5.9% and 11.06%, respectively. Gross total resection, near-total resection, and subtotal resection were achieved in 29.4%, 23.5%, and 47.1% of patients, respectively. Facial nerve function was preserved in 30 of 32 patients (93.8%) without preoperative facial nerve paresis. During a mean follow-up of 2.2 years, 5 patients (14.7%) demonstrated recurrence from the residue in the cisternal portion. All cases of recurrence occurred after STR.

### 3.2. Correlation between MIB-1 and PI and Relevant Clinical Factors

**Figure 2:**
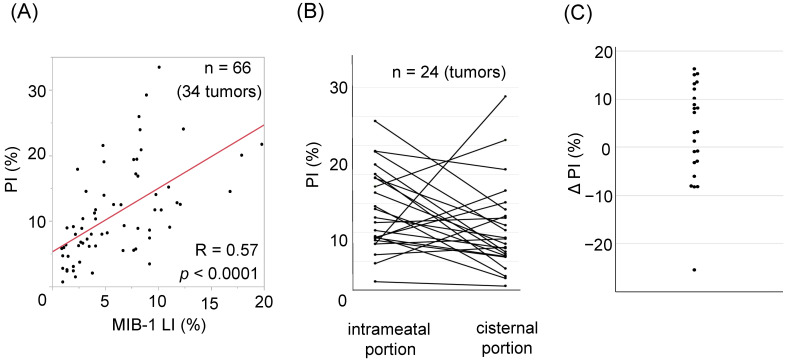
(**A**) Correlation between the proliferation index (PI) and MIB-1 labelling index (R = 0.57, *p* < 0.0001). (**B**) PI are plotted on the left (intrameatal portion) and right (cisternal portion) columns. Specimens from the same tumor are denoted by straight lines. (**C**) Delta PI (intrameatal portion–cisternal portion) are plotted. Note that most tumors demonstrate intratumoral heterogeneity in the PI.

When we evaluated the relationship between MIB-1 LI and PI, a moderate correlation was observed (Figure 2A; R = 0.57, *p* < 0.0001, 95% CI 0.39–0.71). Table 2 presents the results of the univariate analysis of factors associated with recurrence. Age, sex, tumor size, cyst formation, preoperative hearing function, MIB-1 LI, and PI were not associated with an increased risk of recurrence. However, the risk of recurrence was significantly higher after STR (*p* = 0.02).

### 3.3. Intratumoral Heterogeneity of PI

Because the aforementioned preliminary experiment demonstrated that iFC had a high quantitative ability, we next investigated whether there was intratumoral heterogeneity of PI by comparing the intrameatal and cisternal portions. For 24 tumors with a maximum diameter of 2 cm or larger, we obtained specimens from the intrameatal portion and the center of the cisternal portion. Figure 2B demonstrates the differences in PI between those two portions inside the tumor. On plotting the delta PI, we observed a wide variety in PI ranging from −25.48 to 16.3. When we divided these 24 tumors based on the proportion of intrameatal PI to cisternal PI (imPI/cPI ratio), the tumor size was significantly larger in tumors with a higher imPI/cPI ratio (Table 3; 35.4 mm vs. 28.4 mm, *p* = 0.03). In addition, although not statistically significant, there was also a trend wherein preoperative hearing tended to be worse in tumors with a higher imPI/cPI ratio (*p* = 0.06). Facial nerve function was preserved in patients after resection of tumors with a higher imPI/cPI ratio. In two patients (25%), the tumor had a lower imPI/cPI ratio, and they both demonstrated poor facial nerve outcomes (both HB grade III). The actual cPI values of these two tumors were also high (17.2% and 24.0%).

### 3.4. Illustrative Cases

#### 3.4.1. Case 1

**Figure 3:**
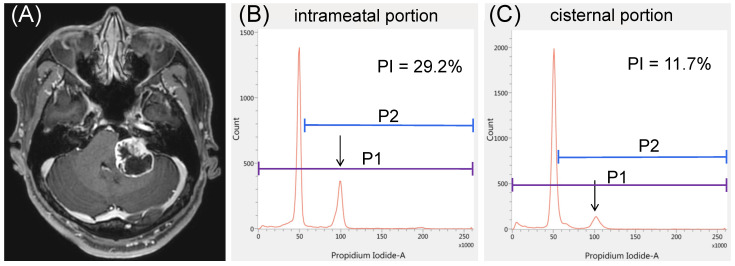
Illustrative case 1. (**A**) A representative case of vestibular schwannoma causing severe hearing loss. The proliferation index (PI) appears to be significantly higher in the (**B**) intrameatal portion than in the (**C**) cisternal portion: 29.2% vs. 11.7%. Arrows represent the peaks of tetraploid cells.

A 48-year-old man with left hearing loss (GR Class V) had cystic VS (Figure 3A) and underwent left suboccipital craniotomy and tumor resection. The results of iFC revealed a higher PI in the intrameatal specimen (Figure 3B, 29.2%) than in the cisternal specimen (Figure 3C, 11.7%). Gross total resection was performed. The patient was discharged with mild facial weakness rated as HB grade II, which resolved within a month. No recurrence was confirmed on MR images obtained 4 years after the surgery.

#### 3.4.2. Case 2

**Figure 4:**
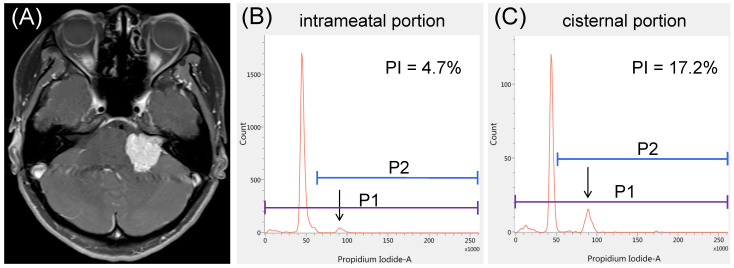
Illustrative case 2. (**A**) A representative case of solid vestibular schwannoma with serviceable hearing. The proliferation index (PI) appears to be significantly lower in the (**B**) intrameatal portion than in the (**C**) cisternal portion: 4.7% vs. 17.2%. Arrows represent the peaks of tetraploid cells.

A 47-year-old woman had a left-sided solid VS causing a sense of imbalance (Figure 4A), but her hearing was normal. The results of iFC showed a higher PI in the cisternal specimen than in the intrameatal specimen (17.2% vs. 4.7%, Figure 4B,C). Owing to severe adhesion to the facial nerve, STR was performed. The patient was discharged with HB grade IV facial weakness on the left side, but her facial nerve function improved to HB grade III after 6 months. No recurrence was observed on MR images at the 2-year follow-up.

## 4. Discussion

The optimal treatment strategy to achieve both long-term tumor control and excellent functional preservation in patients with VS has been a target of debate because the occurrence of these two goals contradict each other. Despite the current advanced surgical techniques and monitoring methods, the rate of good facial nerve function (HB I or II) after gross total resection of large VSs remains at only 65–77% [5,17]. In contrast, the tumor control rate of GKRS for small- to medium-sized previously untreated VS is excellent, reaching 92–98% throughout a 10-year follow-up period. Moreover, the facial nerve preservation rate of the same cohort is 99% or higher. Thus, radiosurgery is currently recognized as the primary treatment for small- to medium-sized VSs [3,18]. However, the treatment outcome of GKRS for larger VSs remains controversial. In a relatively large case series from the last decade that studied GKRS as the primary treatment for large VSs, the tumor control rate dropped to 78.8–98.3% [7,19,20,21,22,23]. These data appear to confirm the importance of surgical resection for tumor control, provided cranial nerve function is reasonably preserved. Although some previous studies have reported that the extent of resection was not associated with VS recurrence after a median or mean follow-up of 3 years [6,24], more than 50% of VSs treated with STR eventually recur if patients are followed up for 7–8 years [9]. Since retreatment—whether by resection [25] or GKRS [26,27]—has reportedly been associated with a slightly elevated complication rate compared to initial treatment, any method to minimize the risk of recurrence from residual tumors would be valuable to balance tumor control and functional preservation. Thus, the present study aimed to explore surgical nuances to improve the quality of STR treatment.

As such, we first explored the usefulness of MIB-I LI in evaluating the proliferative potential of schwannoma cells because the predictive value of MIB-1 LI has not been as firmly established for VS as for meningiomas. A previous study based on the data from 33 patients with VS treated with STR showed that a cut-off value of 1.6% on the MIB-1 LI was useful to predict early recurrence [8]. A recent analysis of 144 patients with sporadic VS found that a cut-off value of 3.5% on the MIB-1 LI was associated with VS recurrence [24]. Another small study of 16 VS cases reported that an MIB-1 LI of 2% or higher was associated with a significantly shorter tumor doubling time [28]. Although one of the advantages of iFC is the high quantitative capability, as shown in our preliminary experiment and previous literature [11,12,13,14,15], our data failed to demonstrate the effectiveness of iFC in assessing the risk of recurrence. This might be because no tumor recurred after gross or near-total resection in our study, suggesting that the extent of resection has greater prognostic significance than the specific proliferative potential in the treatment of VSs.

To improve the quality of life following resection of VSs, preservation of function is preferable and is another topic of debate. A recent study reported that MIB-1 LI ≥ 5% was associated with significantly poor facial nerve outcome in VS surgery [29]. Although this association was not statistically significant in our study, quantitative iFC yielded the same observation as the aforementioned study. It is a common understanding among surgeons that intracanalicular portions of VS can be dissected from the facial nerve more easily than the cisternal portions. These findings appear to indicate that the proliferative potential of the cisternal portion of VS may affect the postoperative facial nerve outcome.

We next investigated whether the location of the residual tumor has an impact on the risk of recurrence by utilizing the high quantification of iFC. Kasbekar, A.V. et al. previously reported that recurrence after STR occurs irrespective of residual tumor sites [30]. On the other hand, Breshears, J.D. et al. recently reported that residual tumor portions within the internal auditory meatus are associated with significantly shorter periods of progression-free survival [10]. They speculated that portions left in the auditory meatus might not be completely devascularized, and, thus, might retain sufficient blood supply to grow in comparison to small residual tumors on the brainstem or cisternal portion of the facial nerve. The present study further revealed that some tumors originally possess more proliferative schwannoma cells within the auditory meatus. This might also be attributable to the uneven distribution of blood supply within a tumor; however, this correlation is difficult to measure. Instead, the present data suggest that a large tumor size and poor preoperative hearing function serve as an index for the higher proliferative potential in the intrameatal portion than in the cisternal portion. We speculate that rapid growth within the narrow bony canal may cause frequent cochlear nerve dysfunction. Nakatomi, H. et al. observed a non-significant favorable trend of lower recurrence rates in patients with serviceable preoperative hearing [9]. However, given that hearing preservation surgery tends to leave small residuals on the cochlear nerve and increases the risk of recurrence, this result appears to be somewhat paradoxical. However, if preoperative good hearing function is a sign of low proliferative ability in the meatus, this is consistent with the present observations. Bloch, D.C. et al. observed no recurrence inside the meatus even after STR [31]. This might be because they utilized a trans-labyrinthine approach for more than 70% of cases. In this approach, removal of the intrameatal tumor and devascularization inside the meatus is normally conducted during surgery, which could contribute to lowering the propensity for recurrence. These reports, along with the current study, advocate for the resection of the intrameatal portion of tumors to reduce the recurrence rate, especially in large tumors causing severe hearing dysfunction. Further studies are required to evaluate the additional effect of resecting the intrameatal portion in planned subtotal resection.

The present study has several limitations. First, this was a single-institution study with a relatively small number of patients, which requires cautious interpretation of the findings when applied to other institutions. Second, it should be reiterated that PI is not directly associated with the recurrence risk of VS, possibly because recurrence does not solely result from the proliferative ability of the resected specimen but rather a mix of factors, such as the proliferative potential of the leftover tumor portion, resection rate, and residual vascularity. In addition, the recurrence risk of histologically benign tumors should ideally be evaluated prospectively with a larger cohort and a follow-up period of 5–10 years. Third, although previous studies have shown that the PI is correlated with MIB-1 and WHO grading of meningiomas [11,13], the evaluation of the MIB-1 LI is subject to inter- and intra-observer biases inherent to the counting method. Finally, although we unified the location to obtain the cisternal specimen as the center of the portion in this study, there might also be heterogeneity inside the large cisternal portion. The heterogeneity in the cisternal portion could have influenced our results, and this issue is related to the intractable limitation of tumor sampling.

It is generally accepted that benign intracranial tumors are histologically homogeneous in any part of the tumor. However, the high quantitative ability of iFC may provide a platform to better understand the biological characteristics of VS. The pursuit of refining a surgical strategy with a better understanding of proliferative potential may ultimately enhance functional outcomes and improve the control rates of large VSs. The results of this study may advocate the resection of the intrameatal portion of large VSs treated with planned subtotal resection, especially in cases of poor preoperative hearing function.

## 5. Conclusions

Our study showed a moderate correlation between the MIB-1 LI and PI. However, neither the MIB-1 nor PI was associated with a significantly increased risk of recurrence in our cohort. On the other hand, the patterns of heterogeneity represented by the imPI/cPI ratio were associated with tumor size. Our data using highly quantitative iFC demonstrated the presence of intratumoral heterogeneity of PI and its relationship with tumor characteristics, such as tumor size and preoperative hearing function. The results of this study may indicate the potential benefit of resection of the intrameatal portion in planned subtotal resection for large VSs with poor preoperative hearing function.

## Figures and Tables

**Table 1:** Patient demographics and characteristics of tumors.

Factor	Value
No. of patients	34
No. of tumors	34
No. of specimens	66
Age (year), mean (range)	57.8 (16–78)
Sex	
Male (%)	13 (38.2)
Female (%)	21 (61.8)
Tumor size (mm), mean (range)	32.3 (14–55)
Tumor type	
Solid (%)	21 (61.8)
Cystic (%)	13 (38.2)
Size of the internal auditory meatus (mm), mean (range)	9.3 (5–20)
Preoperative hearing function (G-R hearing scale) *	
Grade I	6
Grade II	3
Grade III	4
Grade IV	0
Grade V	20
MIB-1 labeling index, mean (range) ^†^	5.9 (0.9–19.8)
PI, mean (range) ^†^	11.06 (0.71–33.45)
Resection rate	
GTR (%)	10 (29.4)
NTR (%)	8 (23.5)
STR (%)	16 (47.1)
Postoperative facial nerve function ^‡^	
Good (%)	30 (93.8)
Poor (%)	2 (6.3)
Recurrence (%)	5 (14.7)

G–R: Gardner–Robertson, GTR: gross total resection, IQR: interquartile range, NTR: near-total resection, PI: proliferation index, STR: subtotal resection. * One patient who was born bilaterally deaf was excluded. ^†^ Mean MIB-1 labeling index and PI were calculated from 66 specimens obtained from 34 patients. ^‡^ House–Blackmann grading scale: grades I and II were classified as good, while grades III–V were considered poor. Two patients with severe facial nerve paresis before surgery were excluded.

**Table 2:** Univariate analysis of factors associated with recurrence (n = 34).

Factor	Recurrence (n = 5)	No Recurrence (n = 29)	*p*-Value
Age (year), mean	58.0	57.8	0.98
Male sex, n (%)	1 (20.0)	12 (41.4)	0.63
Tumor size (mm), mean	36.0	31.9	0.56
Cystic tumor, n (%)	1 (20)	12 (41.4)	0.63
Poor preoperative hearing function, n (%) *	4 (100)	20 (69.0)	0.55
MIB-1 labeling index, mean	7.7	8.4	0.77
PI, mean ^†^	8.1	14.5	0.13
STR	5	0	0.02

IQR: interquartile range, PI: proliferation index, SD: standard deviation. * Poor hearing function was defined as Gardner–Robertson hearing classification scale Class III–V. One patient who was born deaf was excluded from the analysis. ^†^ When PI were evaluated at multiple sites in a single tumor, the highest PI was used for the analysis.

**Table 3:** Univariate analysis of patterns of proliferation index associated with clinical and radiological features (n = 24).

Factor	imPI/cPI Ratio > 1 (n = 15)	imPI/cPI Ratio < 1 (n = 9)	*p*-Value
Age (year), mean	56.1	58.7	0.77
Male sex, n (%)	8 (53.3)	3 (33.3)	0.42
Tumor size	35.4	28.4	0.03
Cystic tumor	4 (46.7)	2 (22.2)	0.39
Size of the internal auditory meatus	8.5	9.7	0.65
Preoperative facial nerve paresis, n (%) *	1 (6.7)	3 (33.0)	0.13
Poor preoperative hearing, n (%) ^†^	13 (86.7)	4 (44.4)	0.06
Good postoperative facial nerve function, n (%)	14/14 (100)	6/8 (75)	0.12

cPI: cisternal proliferation index, imPI: intrameatal proliferation index. * House–Blackmann grading scale: grades I and II were classified as good, while grades III–V were considered poor. ^†^ Poor hearing function was defined as Gardner–Robertson hearing classification scale Class III–V. Two patients with severe facial nerve paresis before surgery were excluded from each group.

## Data Availability

The datasets generated during and/or analyzed during the current study are available from the corresponding author upon reasonable request.
